# Outcomes of 207 totally extraperitoneal hernia repairs using self-fixation mesh

**DOI:** 10.1038/s41598-021-92063-9

**Published:** 2021-06-15

**Authors:** Felipe Girón, Juan David Hernandez, Juan David Linares, Alberto Ricaurte, Andres Mauricio García, Roberto Javier Rueda-Esteban, Lina Rodríguez, Ricardo Nassar

**Affiliations:** 1 Department of Surgery, Hospital Universitario Fundación Santa Fe de Bogotá, 110111 Bogotá, Colombia; 2 School of Medicine, Universidad de los Andes, 111711 Bogotá, DC Colombia; 3 School of Medicine, Universidad del Rosario, Carrera 7 b # 127c - 24, Bogotá, DC Colombia

**Keywords:** Anatomy, Surgery

## Abstract

Inguinal hernia (IH) repair is one of the most common procedures in general surgery around the world. Minimizing postoperative acute and chronic pain without increasing recurrence has been a critical point, giving place to different strategies like self-fixation mesh. The current study aimed to describe a group of patients who underwent IH repair by Totally Extraperitoneal (TEP) technique with self-gripping mesh at a fourth level hospital between 2012 and 2019. Retrospective review of a prospectively collected database including patients who underwent laparoscopic TEP approach with self-fixation mesh for IH repair. Follow up data was obtained at 12, 24, 36, 48, and 60 months post surgical intervention. 207 hernia repairs were performed in 142 patients, with a total of 66 patients with bilateral IH. 10.6% required hospitalization due to either concomitant procedure performed or cardiovascular comorbidities, with a mean hospital stay of 1.6 days. Median and late follow up was up to 5 years. 88.9% of patients complete a year, 86% two years, and 36.7% with a 5 year follow-up. IH repair using the TEP technique and self-fixation mesh showed to be an excellent approach, demonstrating satisfactory results in follow up and complications.

## Introduction

Inguinal hernias (IH) are a very common pathology, originated from a defect in the abdominal wall and/or the inguinal canal, at a collagenous naturally weak region of the abdominal wall referred to as the myopectineal orifice^1,2^. Repair of this defect is one of the most frequently performed procedures in general surgery^3^. Estimated risk of developing IH throughout life is about 27% in men and 3% in women, with a frequency of 10–28 surgical procedures for every 100.000 patients^4^. Gold standard treatment for IH is surgical repair^5^.

Inguinal hernia repairs can be performed by open or minimally invasive approach^6^. First cases of minimally invasive inguinal hernia repair were reported in 1992^7^, with progressive implementation due to its benefits such as decreased postoperative pain and faster recovery with low recurrence rates^2,8^. Multiple studies have found that use of mesh in inguinal hernia repairs reduces the risk of recurrence compared to non-mesh approaches^9–12^. A Cochrane review showed that every 46 hernia repairs using mesh one hernia recurrence was prevented^12^.

Acute and chronic pain represent one of the most important outcomes in postoperative follow up^6^. Minimally invasive procedures offer, as reported in literature, less pain related complications^6^. There are two main laparoscopic techniques used: Trans Abdominal PrePeritoneal (TAPP) and Totally ExtraPeritoneal (TEP)^6,13^. TAPP technique includes laparoscopic exploration of inguinal region and the entire peritoneal cavity, further incision to the overlying peritoneal sheet is performed, reduction of hernial sac and placement of prosthetic mesh against the inguinal wall at the level of preperitoneal space^7^. TEP technique, allows exploration of the myopectineal orifices, dissection and reduction of hernial sac and its content with posterior placement of mesh without entering the abdominal cavity^14^.

Studies comparing both techniques have shown similar complication rates in terms of seroma, scrotal edema, cord swelling, testicular atrophy, urinary and bladder injuries, groin nerve injuries, chronic pain, and recurrence^6,15^. TAPP has a greater risk of visceral injury while extra-peritoneal technique has a greater risk of vascular injury^6^. There are no differences in pain regardless of the minimally invasive technique used, although few studies show better outcomes after TAPP repair 1.15% vs. TEP 3.03%, but with no statistical significance^16,17^. Furthermore, reduction of postoperative pain can be accomplished by optimizing dissection techniques without increasing recurrence rates and decreasing use of mesh fixation devices when possible^18,19^. Besides, to reduce risk of chronic pain associated with suture or tacker fixation, self-adhering, or self-gripping mesh materials (SAMMS) have been developed^19–21^. Overall, there are advantages and disadvantages of both TAPP and TEP procedures^6,22^. There is no statistically significant difference regarding postoperative complications, in terms of recurrence rates and chronic groin pain, hence the decision should be made by the surgical team taking into account all clinical variables and surgical team expertise^6^.

Acute and chronic pain, as well as recurrence, are the most important outcome indicators after inguinal hernia repairs^6^. Therefore, the current study aimed to describe a group of patients who underwent IH repair by TEP technique with self-gripping mesh at a high complexity hospital between 2012 and 2019. Our primary outcome was to evaluate acute and chronic pain after the procedure. Secondary outcomes included establishing the feasibility of mesh insertion during the repair, intraoperative morbidity rates, and a minimum of 1-year recurrence rates.

## Methods

### Study population

After institutional review board (Fundación Santa Fe de Bogota Committee) (IRB) approval and following Health Insurance Portability and Accountability Act (HIPAA) guidelines, a retrospective review of a prospectively collected database was conducted. The study included 142 consecutive patients who underwent laparoscopic TEP approach with self-fixation mesh for inguinal hernia repair between 2012 and 2019. Inclusion criteria were diagnosis of an IH and being over 18 years old. Exclusion criteria included incomplete clinical history, patients intervened in other hospitals, meshes fixated with tackers or any other fixation device, and surgical approach different to TEP. Ethical compliance with the Helsinki Declaration, current legislation on research Res. 008430-1993 and Res. 2378–2008 (Colombia) and the International Committee of Medical Journal Editors (ICMJE) during this research were ensured under our Ethics and Research Institutional Committee (IRB) approval. Informed consent was obtained for the execution of this study.

### Mesh description

SAMMS self-gripping mesh semi absorbable (ProgripTM Mesh, Covidien, New Haven, CT), lightweight (at least 15 × 9 cm) comprised of monofilament polyester and a resorbable polyglactic acid (PLA) gripping system. Mesh weight is 73.0 g/m^2^ (before PLA resorption); 38.0 g/m^2^ (after PLA resorption) and its porosity (pore size: 1.1 × 1.7 mm).

### Surgical technique

Laparoscopic TEP approach was performed in all patients due to surgical team preference and experience. Standardized technique includes a longitudinal midline infra-umbilical incision (12 mm) used to expose the anterior sheath of the abdominal aponeurotic fascia followed by a blunt dissection of the pre-peritoneal space, displacing the abdominal rectus muscles laterally. Dissection balloon or laparoscopic camera is introduced to complete the creation of the pre-peritoneal space, then two midline incisions are created for 5 mm ports, one 3 to 5 cm suprapubic and the other one between the two others. Further dissection of the pre-peritoneal space is carried out to expose the anatomical landmarks, starting the dissection with exposure of the pubic tubercle and the Cooper’s ligament, then the Hesselbach’s triangle and the femoral orifice by clearing the Cooper’s ligament down to the iliac vessels. Internal inguinal orifice (IIO) is also identified, dissected and the cord structures individualized off the hernia sac. Complete reduction of the hernia sac is performed, preserving cord structures. After enough space is created, a 15 X 9 cm self-gripping mesh SAMMS is introduced and placed covering up all the Myopectineal Orifice making sure there is at least a 3 cm flap outside the boundaries of the hernia defect. If the procedure is bilateral, the same actions are carried out, but 1 to 2 cm of overlap between both meshes is always achieved.

### Follow up

Perioperative data included patient demographics and hernia characterization using the European Hernia Society (EHS) inguinal hernia classification and BMI. Intraoperative data included surgical time, intraoperative complications, and additional procedures. The same group of surgeons performed all procedures. Postoperative data included hospitalization time, operative, and postoperative complications: early postoperative pain, hematoma, infection, and reproduction. Follow up data at 8 days, 12, 24, 36, 48, and 60 months was acquired whenever possible. Visual Analog Scale (VAS) was used as a pain assessment tool^14,23^.

### Statistical analysis

Descriptive statistics of all study parameters were provided. Continuous data were summarized by their mean, standard deviation, median, minimum, and maximum. Categorical data were summarized by their frequency and proportion. Associations between quantitative and qualitative variables were compared with a t-test. A p-value < 0.05 was considered significant. Data was analyzed using IBM SPSS Statistics 23 software.

## Results

### Demographic characteristics

There were 142 patients enrolled between May 2012 and January 2019. 28 were women and 114 men. Average age was 59 years old, mean body mass index (BMI) was 25.4 kg/m^2^ (Table 1). Age and BMI were variables that did not show statistically significant differences between both sexes.

**Table 1:** Patients demographics.

	Men		Women	
	Mean	Standard deviation	Mean	Standard deviation
Age (years)^a^	58.57	13.88	62.36	14.03
Weight (kg)	74.15	18.19	65.43	10.44
Height (m)	1.72	0.08	1.60	0.08
BMI (Kg/m^2^)*	25.40	2.96	24.34	3.23

^a^There is no significant difference between sex.

### Procedural characteristics

A total of 207 hernia repairs were performed in 142 patients, with nearly the same number of right and left side hernias, along with 66 patients (46.8%) with bilateral defects. Of all cases, only one patient presented a femoral hernia. As shown in Table 2, 15 (7.25%) cases were considered large or type 3 hernias according to the European Hernia Society (EHS) inguinal hernia classification. However, no other type or larger mesh was used in these cases due to lack of availability. Additionally, on 33 patients (15.9%) other procedures were performed in the same operative time, being the most common cholecystectomy (34%), umbilical (25%), and para-umbilical herniorrhaphy (20%). Intraoperative data is reported in Table 3. Average operative time (including pre-anesthetic preparation) was 73.3 min for unilateral repairs, 10.9 min for bilateral procedures, and 115.9 min for patients that include another procedure besides the hernia repair.

**Table 2:** Hernia classification.

	n	%	
**Laterality**			
Left	40	28.17	
Right	36	25.35	
Bilateral	66	46.48	
**Type**			
Lateral	134	64.73	
Medial	72	34.78	
Femoral	1	0.48	

EHS	> 3	< 3	X
Hernia size^a^	15	104	88

^a^Total of 207 hernias in 142 Patients.

**Table 3:** Intraoperative data.

	n	%
**Intraoperative complications**		
Yes	5	3.5
No	137	96.5
**Additional procedures**		
Yes	33	23.2
No	109	76.8

	Median	
Surgical time^a^	84.26	

^a^There is no significative difference between Additional procedures.

### Complications and follow up

Only 22 (10.6%) patients required hospitalization due to either additional procedure performed or cardiovascular comorbidities, and they had a mean hospital stay of 1.6 days. Two patients presented subcutaneous emphysema during the immediate postoperative period not requiring treatment. Most common intraoperative complication was bleeding of less than 50 cc and was reported in only five cases (4.05%). They did not require rescue maneuvers or conversion to open procedure for bleeding control. One bladder injury was corrected with a single suture followed by bladder catheterization for 2 weeks. Regarding acute postoperative pain, none of patients (0%) in recovery room complained of intense pain (VAS > 4). In the early follow up (8 days post-operative control) 2 (0.97%) patients presented pain (VAS > 4). 4.8% hematomas were reported in 10 patients and 3 seromas were found in patients with large hernias, no surgical site infections were diagnosed, and no early recurrence was reported therefore no patients required an early reintervention (Tables 4 and 5).

**Table 4:** Postoperative qualitative variables.

Follow up	Site infection		Hematoma		Subcutaneous emphysema	
	Immediate	8 days	Immediate	8 days	Immediate	8 days
n	0	0	3	10	2	NA
%	0	0	1.45	4.83	0.97	NA

**Table 5:** Pain control.

Pain	8 days		1 year	
VAS	< 4	> 4	< 4	> 4
n	205	2	182	2
%	99.03	0.97	98.91	1.09
Total	207		184	

Median and late follow up was up to 5 years. 88.9% of patients complete a year, 86% 2 years, and 36.7% with the longest follow-up to 5 years (Table 6). Chronic pain defined as pain after 6 months was diagnosed in 2 (1.09%) patients nevertheless, it was persistent in just 1 patient at 2 years and no patients at 5 years follow up (Table 7). Acute and chronic pain are resumed in Table 5. Recurrence was diagnosed in 1 patient (0.7%) with an associated diagnosis of recurrent colorectal cancer (Table 8).

**Table 6:** Follow-Up.

Follow up	6 months	1 year	2 years	3 years	4 years	5 years
**Total Hernias**						
207	207	184	178	138	105	76
%	100,0	88,9	86,0	66,7	50,7	36,7

**Table 7:** Pain control follow-up.

Pain	6 months	1 year	2 years	3 years	4 years	5 years
n	2	2	1	1	0	0
%	0,97	1,09	0,56	0,72	0	0

**Table 8:** Recurrence.

Recurrence	Yes	No
n	2^a^	205
%	0.96	99.04
Total	207	

^a^Both recurrences were diagnosed in 1 patient (0.7%) with an associated diagnosis of recurrent colorectal cancer.

## Discussion

Inguinal hernia is a complex surgical pathology, with poor consensus in some aspects of its surgical repair, such as the best approach, mesh type and predisposing factors of postoperative pain^6^. Minimally invasive surgery (MIS) has demonstrated to offer an equally safe and cost-effective option for groin hernia repair even in large defects compared with open procedures^6^. Prosthesis reinforcement of the myopectineal orifice has been constituted as a gold standard approach that shows an important recurrence reduction over time^6,24^. Many strategies aiming for pain reduction without altering recurrence rates have been elucidated, being non-invasive fixation methods more frequently suggested as feasible^4,17,21,24–28^.

Mean operative time was 73.3 min in unilateral repairs, 101.9 min in bilateral procedures (including pre-anesthetic preparation) and 115.9 min in IH repair and concomitant procedures as well, which are similar and even lower to median rates 102.12 min in bilateral and 96.63 min for unilateral repairs, in other studies using this technique with and without the same prosthesis^28,29^. Some authors already describe mesh placement time around 5 min for laparoscopic approaches but in our study mesh placement time was not measured^4,30^.

Common perioperative complication rates in inguinal hernia procedures are low, Stavert et al. reported, in 780 laparoscopic inguinal hernia repairs, a complication rate of 0.13–1.67% being seroma (1.41%), hematoma (1.41%) and surgical site infection (SSI) (0.13%) the most frequently found^31^. Results similar to those were found in our study with a slightly higher presence of seroma but no presence of SSI (Seroma 2.1%, hematoma 1.45% and SSI 0%)^21,32,33^. In terms of SSI, adding clean contaminated procedure to hernia inguinal repair still remains controversial due to believed aggregated risk, nevertheless, as Quezada et al. described in 21 patients who underwent concomitant laparoscopic hernia repair and cholecystectomy, we found lower incidence of SSI^34^. The lack of surgical site infection in our study likely is associated with the performing of the clean procedure first as well as the cavity separation accomplished by TEP technique, nevertheless no clear association can be established and should be evaluated in further studies^34^.

Most patients did not require further post-operative care, while 23 patients needed hospitalization, often related to additional procedures performed (cholecystectomy, umbilical hernia). No pneumomediastinum, pneumothorax, or bowel obstructions related to laparoscopic methods were observed, as Gass et al. described due to the avoidance of abdominal cavity access through the TEP method^12,22^.

Seeking reduction in recurrence rates and improvement of postoperative acute and chronic pain different non-invasive mesh fixation methods have been described, ranging from self-gripping meshes, preformed prosthetics to biological adhesives^24^. Theoretical advantages of self-gripping meshes arise from its adhesive mechanism that allows a safe fixation even in critical areas such as pain and doom triangles^27,31,35^. Nonetheless, some concerns regarding mesh maneuverability remain unsolved^35,36^. Other non-invasive techniques like fibrin glue are less cost-effective, due to the high cost of biological glue despite its safeness in critical areas^37^. Conversely, invasive fixation methods disrupt tissue surface, require supplementary devices, leave additional objects in the cavity and as Moreno-Egea et al. showed, in a randomized clinical trial, fixation in TEP offers no advantages and increases procedure costs over non-fixation^38^.

Recurrence rate, as one of the milestones in hernia surgery follow up, was low (0.7%), equivalent to other self-fixation mesh experience rate (< 1%) reported by Stavert et al. Furthermore, recurrence rate found is lower compared to other studies matching different surgical techniques (open, TAPP, TEP) and fixation methods (Invasive, non-invasive, not fixation) with reported rates that may vary between 1 to 15%^4,21,27,30,32,33,39–42^. As reported in literature analysis of a non-invasive fixation (self-fixation mesh) technique in our population showed low morbidity preoperatively and in the late follow up^16,22^. No mortality is reported concerning the surgical procedure itself.

Regarding acute postoperative pain, no patients reported severe pain (VAS > 4). 2 (1,09%) patients remained with pain at 1 year consult and one of them (1.3%) persisted with pain in the late follow-up (persisted at 3 years but remitted at 4 years ). In respect of pain persistence in the late follow-up we had two scenarios, the patient whose pain remained at the 1 year consult, was described as a tingling sensation that occurred during physical activity and remained a few hours after, did not require analgesics and faded before the 2 year control. On the other hand, the patient whose pain persisted for 3 years, who had been diagnosed with left hernia 2 months prior to the surgery and whose initial symptom was ocasional pulling pain in the inguinal region, remained symptomatic for 40 months after the surgery but did not felt the need to use analgesia and referred the pain as insignificant. Both scenarios presented are congruent with reported results in literature, Mitura et al. in a study with 1647 patients found that greater pain was reported in patients more active professionally or those who performed heavily manual tasks, as our cases^31,43^. In respect of pain, results of our study are affected because a small percentage of patients completed a long time follow up (5 year evaluation), chronic pain is lower compared to similar studies, showing rates of 3.5% (with mesh) and 2.9% (without mesh)^21,29,36^.

The main limitation of this study is that it is not a comparative study. The aim was to show the experience in this approach with a long follow up. Although, studies have appeared in recent years regarding this topic, more randomized controlled and with a strict follow up studies are needed to define and conclude if the self-fixation mesh is the best option to decrease overall pain without a compromise in recurrence rate for IH repair in an MIS approach.

## Conclusion

Inguinal hernia repair using the TEP technique and self-fixation mesh showed to be an excellent approach, avoiding fixation devices usage and demonstrating satisfactory results in perioperative complications, pain control, reproduction, and chronic complications. Further investigation is required to confirm if self-fixation is the best method to assure less recurrence and chronic pain, in contrast, to no fixation at all, and to describe in greater detail if outcomes are in any way affected by hernia type or location, even with experienced surgeons.

## Data Availability

The datasets generated during and analyzed during the current study are available from the corresponding author on reasonable request.
